# Combating Malnutrition: Nutrient and Energy Composition of Locally Formulated Ready-to-Use Therapeutic Foods for Children

**DOI:** 10.3390/ijerph22121845

**Published:** 2025-12-10

**Authors:** Amina Sa’id Muhammad, Eridiong Ogbonna Onyenweaku, Kamaluddeen Babagana, David Sale Danjuma, Raymond Nabem Beba

**Affiliations:** 1 Nutrition Unit, Department of Biochemistry, Faculty of Basic Medical Science, College of Health Sciences, Bayero University Kano, Kano P.M.B. 3011, Kano State, Nigeria; asmuhammad.bch@buk.edu.ng (A.S.M.); kbabagana.bch@buk.edu.ng (K.B.); raymondbeba355@gmail.com (R.N.B.); 2 Nutrition and Dietetics Department, Aminu Kano Teaching Hospital, Kano P.M.B. 3452, Kano State, Nigeria; 3 Food Evolution Research Laboratory, School of Tourism & Hospitality, College of Business & Economics, University of Johannesburg, Johannesburg 2092, South Africa; 4 Department of Public Health, Faculty of Allied Health Sciences, University of Calabar, Calabar P.M.B. 1115, Cross River State, Nigeria; debsydavidson@gmail.com

**Keywords:** Ready-to-Use Therapeutic Food (RUTF), malnutrition, proximate analysis, sensory evaluation, child nutrition, Nigeria

## Abstract

**Background**: Nigeria faces a severe child malnutrition crisis, with approximately 1 million severe cases reported for 2025. This burden positions Nigeria among the top countries globally for stunting and wasting in under-fives, exacerbated by factors like food insecurity, flooding, and conflict—particularly in the northern part. This study investigated the development and assessment of Ready-to-Use Therapeutic Foods (RUTF) produced from locally sourced ingredients in Kano State, Nigeria, targeting child malnutrition. **Methods**: Three distinct RUTF formulations were prepared using rice, wheat, groundnut, and soybean, with raw materials purchased from local markets and processed into blends. Proximate, vitamin (A, C, and E), and mineral (zinc, iron, potassium, magnesium, calcium, and sodium) compositions were measured following the Association of Official Analytical Chemists’ (AOAC) standard procedures. Sensory evaluation using a 9-point hedonic scale assessed taste, aroma, flavour, appearance, and overall acceptability. **Results**: Notable variations were observed among the samples. Blend A exhibited the highest energy (563.08 kcal/100 g), carbohydrate (46.57%), fat (35.84%), and vitamin E (9.29 mg/100 g) content. Blend B was highest in protein (16.71%), iron (2.40 mg/100 g), calcium (21.05 mg/100 g), and vitamin A (15.89 µM). Blend C contained the most potassium (61.65 mg/100 g) and vitamin C (11.70 mg/100 g), with moderate levels of other nutrients. Sensory ratings showed no significant (*p* < 0.05) differences among the parameters. **Conclusions**: The nutrient composition and acceptability of RUTF blends suggest that affordable, effective dietary solutions can be produced using local crops. These findings support the potential for locally formulated RUTFs to contribute to reducing child malnutrition in low-resource settings with further enhancements.

## 1. Introduction

Malnutrition remains one of the most urgent and persistent global public health challenges, particularly among children under five years of age. According to the World Health Organization, undernutrition contributes to nearly half of all deaths in this vulnerable age group, with far-reaching consequences on survival, physical growth, cognitive development, and long-term productivity [1]. Global estimates suggest that stunting, wasting, and micronutrient deficiencies continue to affect millions of children, with the highest burden concentrated in low- and middle-income countries [2]. These forms of undernutrition not only increase susceptibility to infectious diseases but also perpetuate intergenerational cycles of poor health outcomes [3].

In sub-Saharan Africa, particularly Nigeria, the prevalence of childhood malnutrition remains alarming despite decades of nutritional interventions. The 2023/2024 Nigeria Demographic and Health Survey reported that 40% of children under five are stunted, 8% are wasted, and 27% are underweight, reflecting a sustained burden of undernutrition [4]. These statistics place Nigeria among the countries with the highest number of malnourished children globally, underscoring the urgent need for effective, accessible, and sustainable solutions [5].

Ready-to-Use Therapeutic Foods (RUTFs) have been introduced as one of the most effective innovations for addressing severe acute malnutrition (SAM). These nutrient-dense, lipid-based pastes are designed to deliver high energy and protein content, along with essential vitamins and minerals, without requiring refrigeration or cooking [6]. Their stability, ease of distribution, and suitability for both inpatient and community-based treatment programs make them uniquely valuable in emergency and resource-limited contexts [7]. Evidence shows that RUTF significantly improves recovery rates and reduces mortality among children with SAM, thus becoming a cornerstone of child nutrition programs [8].

Despite these successes, challenges remain regarding the sustainability and scalability of RUTF programs in Nigeria. Most therapeutic foods currently used are imported, creating issues of affordability, supply chain disruptions, and dependence on external donors [2]. The high costs of imported products limit availability, especially in rural and low-income settings, while cultural unfamiliarity sometimes reduces compliance [9]. According to the UNICEF Supply Division [10], global RUTF supply chains remain vulnerable to rising costs, production delays, and distribution constraints, which further compound challenges in countries like Nigeria. These limitations point to the need for developing locally produced alternatives that are not only nutritionally adequate but also affordable, culturally acceptable, and supportive of the domestic agricultural sector [11].

Recent research has demonstrated that locally available cereals, legumes, and plant-based proteins can be effectively utilized in RUTF formulations. For instance, Adewumi et al. reported that Bambara groundnut combined with Moringa oleifera leaf protein complex produced nutritionally rich, stable formulations [12]. Similarly, Umar and Orishagbemi [13] highlighted that cereal–legume samples yielded products with acceptable nutrient profiles and sensory qualities comparable to standard RUTF. Studies conducted in Nigeria and Ghana further showed that formulations based on maize, soybeans, groundnut, and wheat are not only cost-effective but also culturally familiar, which enhances acceptability among caregivers and children [14,15]. Moreover, Ishaq et al. emphasized the feasibility of the community-level production of RUTFs, suggesting that decentralized approaches could address availability gaps [16].

However, gaps remain in the systematic evaluation of locally formulated RUTFs in terms of nutrient adequacy and sensory acceptability. While energy and macronutrient requirements are often prioritized, micronutrient fortification and bioavailability, as well as cultural preferences regarding taste and texture, require further attention to ensure effectiveness and compliance [5]. Addressing these gaps is particularly important in Nigeria, where regional variations in food availability and cultural diets may influence both formulation and acceptance.

The present study, therefore, aims to contribute to this growing body of knowledge by formulating and evaluating RUTF blends from locally available food resources in Kano State, Nigeria. Specifically, this study investigates proximate composition, mineral, and vitamin profiles and sensory properties of the formulations. By doing so, it seeks to provide evidence for the feasibility of affordable, culturally acceptable, and nutritionally adequate RUTFs that could support efforts to reduce the burden of severe acute malnutrition in Nigerian children.

## 2. Materials and Methods

### 2.1. Study Design

This was an experimental laboratory study involving the formulation and evaluation of three Ready-to-Use Therapeutic Food (RUTF) blends prepared with locally available ingredients; this study was carried out from April to June 2025. Nutritional (proximate, mineral, and vitamin) composition and sensory properties were analysed to determine the adequacy of the formulations.

### 2.2. Location and Sample Size

This study was conducted in Kano State, Northwestern Nigeria, a major commercial and agricultural hub with active local markets where staple crops such as rice, wheat, groundnut, and soybean are widely available. Laboratory analyses were performed at Bayero University Kano (BUK), which provides well-equipped facilities for food and nutrition research. Raw materials were sourced from Brigade Market, Nassarawa Local Government Area, and identified at the University Herbarium with assigned voucher numbers: rice (0289), wheat (0398), groundnut (0405), and soybean (0506).

### 2.3. Ingredients and Equipment

The ingredients included soybeans (*Glycine max*), groundnut (*Arachis hypogaea*), brown sugar, soya oil, baobab (*Adansonia digitata*), date palm (*Phoenix dactylifera*), wheat (*Triticum aestivum*), and white rice (*Oryza sativa*). All ingredients (soybeans, groundnut, soya oil, baobab, date palm, wheat, and rice) are widely grown and/or consumed in Kano State and the northwestern region, featuring in daily diets and traditional foods. The brown sugar used is locally processed from sugar cane locally grown in the region, not the refined one.

Key equipment included the following: muffle furnace (Carbolite CWF 1200; 15 Par-sons Ln, Hope Rd, Hope Valley S33 6RB, UK), Soxhlet extractor (Buchi Labortechnik AG, E-816; Meierseggstrasse 40, 9230 Flawil, Switzerland), oven (Genlab MINO/100; Tanhouse Lane, Widnes, Cheshire, WA8 0SR, UK), spectrophotometer (JENWAY 6705 UV/Vis; Bea-con Road, Stone, Staffordshire ST15 0SA, UK), weighing balance, measuring cylinders, and beakers. Analytical grade reagents used included hydrochloric acid (HCl), nitric acid (HNO_3_), sodium hydroxide (NaOH), and n-hexane.

All analyses were carried out in replicates to ensure reproducibility. Equipment was calibrated before use, and reagents were of analytical grade. Samples were stored in airtight containers at room temperature to prevent contamination or spoilage before analysis.

### 2.4. Formulation of RUTF Blends

The raw materials were sorted, washed, and sun-dried. Each was processed into flour or paste:

1. Soybean flour: fermented for 24 h, dehulled, toasted, and ground.
2. Groundnut paste: roasted for 20 min and ground.
3. Rice and wheat: decorticated, sun-dried, and milled into powder.
4. Brown sugar: milled into powder.

Three RUTF formulations were developed by varying ingredient proportions (Table 1).

The choice of the three blends is nutritionally appropriate to formulate an energy-dense paste that can be compared to the WHO/UNICEF RUTF targets (520–550 kcal/100 g; 10–12% of energy from protein; 45–60% from fat) while using Nigerian crops. These crops/ingredients (soybean, groundnut, rice, wheat, dates, baobab, soya oil, and brown sugar locally processed from sugarcane) are locally available and affordable, which reduces production costs, import dependence, and supply disruptions.

Soybeans (30% in all blends) act as a primary protein source replacing milk powder in the standard RUTF, and they are a high-quality plant protein rich in lysine and complement cereal proteins well.

Soya oil (15–25%) is a major contributor to energy density and essential fatty acids (linoleic and alpha-linolenic acids), improving the absorption of fat-soluble vitamins and providing the smooth, spreadable texture required for RUTF.

Peanuts (25% in Blend A only) serve as a traditional RUTF base that adds energy, protein, flavor, and texture. It is retained in one option for cost and acceptance where aflatoxin control is reliable.

Date palm (28% in Blend A) is a natural sugar that enhances palatability as it contains potassium, polyphenols, and fiber, and it keeps water activity low because of its low-moisture content.

Brown sugar (28% in Blends B and C) acts as a low-moisture energy source that supports shelf stability when moisture control is challenging.

Cereals (20% rice in blend B; 15% wheat in blend C) provide structure, bulk, and a bland carrier that moderates beany notes, reduces cost, and complements soy amino acids (soy is lysine-rich; cereals contribute methionine).

Baobab (2% in all blends) is naturally high in vitamin C, which enhances non-heme iron absorption, in addition to calcium, potassium, and organic acids that brighten flavor. Small inclusion improves micronutrient profiles without affecting texture.

The season and harvest stage can meaningfully shift some nutrients, like moisture, certain vitamins, and oil profiles, while most minerals and bulk macronutrients on a dry-matter basis remain relatively stable.

Locally developed ready-to-use therapeutic food (RUTF) formulations show meaningful cost advantages compared to standard, widely available products. Studies have documented cost savings ranging from 14% to 52% when alternative formulations replace standard RUTFs [17]. This substantial variation reflects differences in ingredient sourcing, production scale, and formulation complexity across different local initiatives.

### 2.5. Proximate Analysis

Proximate compositions (moisture, ash, crude protein, crude fat, crude fibre, and carbohydrate by difference) were determined using standard AOAC methods [18,19]:

- Moisture content: Oven-drying at 105 °C to constant weight.
- Ash content: Dry ashing in a muffle furnace at 550 °C.
- Crude protein: Kjeldahl method using a 6.25 nitrogen-to-protein conversion factor.
- Crude fat: Soxhlet extraction with petroleum ether.
- Crude fibre: Sequential acid–alkali digestion.
- Carbohydrate: Determined by the following difference:

%CHO = 100 − (% moisture + % ash + % protein + % fat + % fibre).

### 2.6. Determination of Micronutrient Composition

Minerals (calcium, magnesium, iron, zinc, sodium, and potassium) were determined using Atomic Absorption Spectrophotometry (AAS) after dry ashing and dissolution in 1% HCl. Vitamins (A, C, and E) were determined using High-Performance Liquid Chromatography (HPLC) following standard procedures.

### 2.7. Sensory Evaluation

Twenty semi-trained Panelists (comprising mother–child pairs) were carefully selected among the Female Nutrition and Dietetics Students within the Nutrition Unit of Biochemistry Department, Bayero University, Kano. The mothers evaluated the sensory attributes (taste, texture, aroma, colour, and overall acceptability) of the three RUTF blends using a 9-point hedonic scale (1 = extremely dislike; 9 = extremely like). Subsequently, the samples were then presented to the children, and their preferences for each sample were interpreted by their mothers; the average score was recorded as overall acceptability. The samples were presented in coded, randomized order under identical conditions. Panellists rinsed their mouths with water between evaluations. The panellists were not allowed to discuss among themselves.

Ethical clearance was obtained from the Health Research Ethics Committee of the State Ministry of Health; the panellists were provided with informed consent forms, which they signed, and this study’s purpose, risks, and benefits were explained to them to ensure confidentiality and voluntary participation. The sensory evaluation exercise was carried out in a sensory laboratory at the department, which has individual testing booths designed to eliminate distractions and biases.

The samples were presented in coded, randomized order under identical conditions to the mothers. They evaluated the samples based on the sensory attributes (taste, texture, aroma, colour, and overall acceptability) of the three RUTF blends using a 9-point hedonic scale (1 = extremely dislike; 9 = extremely like). Panellists rinsed their mouths with water between evaluations, and they were not allowed to discuss among themselves. Subsequently, the samples were then presented to the children (6–59 months), and their preferences for each sample were interpreted by their mothers as they observed their facial expressions and body language to record their reactions. This method was adopted from a study by Ciliberto et al. [20]

### 2.8. Statistical Analysis

Data were analysed using IBM SPSS Statistics (Version 25.0). All dependent variables were subjected to One-Way Analysis of Variance (ANOVA) to determine if significant differences existed between the three RUTF blends (A, B, and C). The assumptions of normality and homogeneity of variance were checked before analysis.

Effect sizes were reported as partial eta-squared (η^2^), where 0.01, 0.06, and 0.14 represent small, medium, and large effects, respectively. Where the ANOVA indicated a significant main effect (*p* < 0.05), post hoc comparisons were conducted using the Bonferroni correction to control for Type I errors across the three pairwise comparisons. Consequently, a stricter significance threshold of *p* < 0.017 (0.05/3) was applied for all post hoc tests. The results are presented as Mean ± Standard Deviation (SD), along with the F-statistic and degrees of freedom (F(df_between_, df_within_)).

### 2.9. Ethics

Ethical approval was sought for and obtained from the Health Research Ethics Committee of the Ministry of Health, Kano State, with the following approval number: NHREC/17/03/2018. The document is dated 23 March 2025.

## 3. Results

### 3.1. Proximate Composition of the RUTF Blends

The proximate composition of the RUTF blends is presented in Table 2. The results show that, among the three blends, Blend A had the highest energy value (563.08 kcal) while Blend B had the lowest energy value of 503.67 kcal. Blend A also recorded significantly (*p* < 0.05) higher carbohydrate content of 46.57% when compared to the others. Blend B had the highest protein content (16.71%) and ash (3.76%), which were significantly higher than the values recorded by Blend A. Blend B also had the lowest fat content of 28.15%, which differed significantly from that of Blends A and C.

### 3.2. Sensory Evaluation of the RUTF Blends

Table 3 shows the results for the sensory evaluation of the RUTF blends using the 9-point hedonic scale. The results are the calculated mean scores recorded by the 20 panellists who carried out the evaluation. No significant difference (*p* > 0.05) is observed between all three blends in terms of their taste, aroma, flavour, and overall acceptability. Notably, Blend C differs significantly (*p* < 0.05) from Blend A and B in terms of appearance, with a lower mean score of 5.73 ± 2.56.

### 3.3. Vitamin Content of the RUTF Blends

The vitamin analysis revealed significant differences among the three RUTF blends for vitamins A, C, and E (*p* < 0.05) as seen in Table 4. Blend B contained the highest level of vitamin A (15.89 ± 0.37 µM), significantly greater than Blend C (12.86 ± 0.15 µM) and Blend A (9.13 ± 0.02 µM), indicating that Blend B may better support visual function and immune health in target populations. For vitamin C, Blend C delivered the highest concentration (11.70 ± 0.14 mg/100 g), which was statistically higher than both Blend B (9.57 ± 0.24 mg/100 g) and Blend A (7.39 ± 0.10 mg/100 g). Elevated vitamin C contents in Blend C could enhance antioxidant intake. Regarding vitamin E, Blend A presented the highest content (9.29 ± 0.19 mg/100 g), which was significantly higher than Blend B (8.16 ± 0.19 mg/100 g) and Blend C (8.59 ± 0.12 mg/100 g). Vitamin E is essential for cell membrane protection and immune function, indicating Blend A’s potential advantages in reducing oxidative stress.

### 3.4. Mineral Composition of the RUTF Blends

Statistically significant differences (*p* < 0.05) were observed among the three RUTF blends for all analysed minerals, as seen in Table 5. Blend B demonstrated the highest concentrations of iron (2.40 ± 0.02 mg/100 g) and calcium (21.05 ± 0.04 mg/100 g), suggesting that it provides better support for haematological function and bone health compared to the other blends. Blend C exhibited a remarkable potassium level (61.65 ± 1.43 mg/100 g), far exceeding the values found in Blends A and B, which may offer a key advantage for maintaining electrolyte balance. In contrast, Blend A contained the highest magnesium content (1.07 ± 0.04 mg/100 g) and moderate levels of other minerals. For zinc, Blend B also led (0.39 ± 0.00 mg/100 g), while Blend A contained the lowest sodium (6.04 ± 0.00 mg/100 g), which may be preferable for specific dietary requirements. Each blend displayed a unique mineral profile: Blend B as the richest in iron, calcium, and zinc; Blend C as the highest in potassium; and Blend A as notable for magnesium and lower sodium content.

#### Composition of the Local Formulation Versus Standard RUTF

The nutrient composition analyses in Table 6 showed that the protein contents of the formulated blends (A—13.56%, B—16.71%, and C—14.62%) were found to be higher than the standard WHO RUTF of 10–12%. Energy (kcal/100 g) for blend A (563.08 kcal/100 g) was found to be higher than the WHO RUTF standard, while that of blend B (503.67 kcal/100 g) was lower than the standard, and blend C (528.98 kcal/100 g) was within the standard WHO RUTF range of 520–550 kcal/100 g. The fat values were 35.84% for blend A and 28.15% for blend B, and the blend C sample recorded 34.10%, which were all lower than the WHO RUTF (45–60%). With regard to the mineral analyses, the formulated blend samples apparently had lower values compared to those of the standard.

## 4. Discussion

This study formulated and evaluated Ready-to-Use Therapeutic Foods (RUTFs) from locally available Nigerian ingredients, focusing on their nutritional composition, vitamin and mineral content, and sensory acceptability for the management of severe acute malnutrition (SAM). The findings showed distinct differences across the three formulations, with each demonstrating unique strengths. This highlights the potential of locally produced RUTF to serve as effective alternatives or complements to imported products.

In terms of proximate composition, the formulations varied in their contributions of energy, protein, fat, and fibre, reflecting how ingredient selection influences overall nutrient balance. One blend was richer in protein and ash, and another was characterized by higher energy and fat, while the third provided greater fibre and moisture content. Similar variations have been reported by Nwankwo et al. [14], who observed comparable energy and protein values in locally produced formulations. Ishaq et al. [16] also found protein and fat levels within a similar range, while Edafioghor et al. [11] reported lower energy densities, suggesting that formulation design plays a crucial role in achieving nutrient adequacy. These findings support the feasibility of formulating RUTF that aligns with WHO recommendations using locally sourced crops.

The mineral composition also differed substantially across the blends. One formulation was richest in iron, zinc, and calcium, while another was distinguished by its higher potassium content. These differences mirror the variability observed in studies such as Adewumi et al. [12], where snack bars formulated with Bambara groundnut and Moringa provided minerals comparable to RUTF, though potassium was lower. In contrast, Edafioghor et al. [11] reported suboptimal levels of zinc and potassium, underlining the importance of ingredient selection in mineral adequacy. Umar and Orishagbemi [13] further emphasized that local crop profiles significantly determine mineral contributions. The present study, therefore, suggests that locally formulated RUTF can be tailored through formulation adjustments to target specific nutritional deficiencies. The variations observed demonstrate the feasibility of producing RUTF blends optimized for particular health needs, such as anaemia or electrolyte management. Collectively, all RUTF blends exhibited mineral levels appropriate for addressing micronutrient gaps in children at risk of malnutrition, with potential for further enhancement through ingredient and processing choices.

The vitamin profiles also revealed complementary strengths across the formulations. Overall, each RUTF blend excelled in specific vitamins: Blend B in vitamin A, Blend C in vitamin C, and Blend A in vitamin E. This reflects patterns reported by Yakubu et al. [15], who highlighted the variability of vitamin content in local RUTF compared with WHO standards. Edafioghor et al. [11] also demonstrated that vitamin A and E levels in their formulations fell within recommended ranges, though vitamin C information was limited. These findings indicate that while no single local formulation provides complete vitamin coverage, combining or adjusting formulations can enhance micronutrient adequacy. These variations may allow for targeted nutritional support depending on priority micronutrient needs. The results suggest that the formulation process can be adjusted to optimize vitamin content, contributing to the design of more effective and nutrient-rich therapeutic foods for children at risk of undernutrition. Some local sources of vitamin A, C, and E include carrots, green leafy vegetables, and nuts, respectively.

In terms of sensory acceptability, all blends were generally well received, with one being most preferred overall, another ranking higher for taste, and another for aroma. These results are consistent with Nwankwo et al. [14], who reported that locally formulated RUTF achieved acceptability comparable to imported alternatives. Ishaq et al. [16] similarly observed that formulations with locally familiar ingredients were well accepted, while Yakubu et al. [15] noted that although the WHO standard RUTF remained the most preferred, local formulations were still considered acceptable. This suggests that culturally familiar ingredients may improve compliance, an important factor in therapeutic feeding. Readily available and easily accessible ingredients also make these locally formulated RUTFs more acceptable.

Taken together, the findings demonstrate the feasibility of formulating nutritionally adequate and acceptable RUTF using Nigerian indigenous crops. Previous studies have shown that locally sourced formulations can reduce production costs while maintaining nutritional quality [9]. Nwankwo et al. [14] similarly highlighted that local production improves sustainability and accessibility. Systematic reviews have also confirmed that both standard and locally produced RUTFs are effective in improving recovery outcomes among malnourished children [7] and are cheaper than standard RUTFs. It is worth noting that the RUTFs contribute a significant percentage to the recommended daily intakes for both macro- and micronutrients.

Food fortification policies in Nigeria play a critical role in addressing childhood malnutrition, particularly micronutrient deficiencies that contribute to stunting, wasting, and underweight among children [23]. Large-scale food fortification (LSFF) initiatives mandated by the government, such as the addition of vitamin A to edible oil, iron to wheat flour, and iodization of salt, have demonstrated significant reductions in micronutrient deficiencies in the target populations. These policies are considered the most effective and scalable interventions, reaching diverse population groups with proven health impacts. Recent reviews [23,24,25] and monitoring efforts show that despite challenges of implementation and coverage, fortification remains vital as part of a multi-faceted national strategy targeting child malnutrition. On the other hand, vitamin supplementation (particularly vitamin A) remains a key intervention to address micronutrient deficiencies among children in Nigeria. Despite proven benefits, challenges such as supply chain issues, limited access in rural areas, and periodic shortage of vitamin capsules have hindered optimal coverage and effectiveness of supplementation programmes [26]. As a result, many children still remain vulnerable to malnutrition and its associated health risks. The introduction of these newly developed Ready-to-Use Therapeutic Foods (RUTF), specifically formulated to meet local preferences and micronutrient requirements, offers a sustainable solution [11]. These RUTFs can bridge gaps left by supplementation alone, providing not only vitamins but also energy and other essential nutrients in an accessible, palatable form suitable form.

The locally formulated RUTF blends demonstrated higher protein and carbohydrate contents than the WHO standard, with protein ranging from 13.56% to 16.71% compared with the recommended 10–12% and carbohydrates exceeding the 30–40% benchmark, suggesting good potential to support catch-up growth. However, fat levels in all blends (28.15–35.84%) were below the WHO target of 45–60%, which partly explains why only blend A met and exceeded the energy specification, while blend B fell short, and blend C just met the lower bound of the 520–550 kcal/100 g standard. These findings align with other local RUTF development studies, such as Edafioghor et al. [15], who reported that inadequate fat contributions in cereal–legume formulations tended to lower energy density despite acceptable protein levels. Similarly, the nutrient evaluation of cereal- and legume-based RUTFs by another group found that failure to reach the WHO-recommended fat ranges resulted in energy values below 520–550 kcal/100 g, even when protein and carbohydrate levels were satisfactory. Consistent with prior work, mineral contents in the present blends were generally lower than WHO RUTF specifications, confirming reports that locally sourced RUTFs often require additional mineral premixes or fortification to match standard micronutrient profiles [13,17]. Despite the low Ca and Mg content, the Ca-Mg ratios of the RUTF blends are quite high, and this may cause poor absorption of magnesium [27]. Consequently, the inclusion of a micronutrient-rich plant food, as well as possible fortification, will go a long way in enhancing the mineral profile and subsequent absorption. Furthermore, a randomized control trial in Indonesia reports that RUTFs based on local recipes are as efficacious and have higher acceptability than standard peanut-based RUTFs [28].

A strength of this study is the detailed evaluation of proximate, vitamin, mineral, and sensory attributes, providing a holistic view of the formulations. However, the absence of amino acid profiling, which is critical for assessing protein quality [12], is a limitation. Furthermore, clinical trials assessing recovery outcomes in children are needed to validate the practical effectiveness of these formulations.

This study demonstrates that RUTF made from locally available Nigerian ingredients can meet nutritional requirements while maintaining sensory appeal. Although one formulation—Blend B emerged as the most acceptable overall in terms of sensory properties, others provided unique strengths in specific nutrients (Blend A recorded significantly higher energy value). These results suggest that combining or optimizing local formulations may produce balanced, cost-effective, and culturally appropriate therapeutic foods for the management of severe acute malnutrition in Nigeria.

## 5. Conclusions

The locally formulated RUTF met the energy requirements of more than 500 kcal per 100 g and contained an appreciable amount of macro- and micronutrients. The appearance, taste, flavour, and overall acceptability of the blends were commendable, highlighting the potential for local production to effectively combat severe acute malnutrition among children under five using locally available plant-based crops in Northern Nigeria. Consequently, this study highlights the promising potential of Ready-to-Use Therapeutic Foods (RUTFs) formulated from locally available ingredients to address malnutrition in children—a vulnerable population. These findings align with global evidence supporting local production as a cost-effective, sustainable approach to improving nutritional interventions for vulnerable children. While this study provides comprehensive compositional data, future research should include clinical trials to validate health outcomes and explore protein quality in depth. RUTF formulations based on nutrient strengths may enhance therapeutic efficacy. Ultimately, locally formulated RUTF can play a critical role in combating malnutrition in resource-limited settings, contributing to improved child health and survival.

## Figures and Tables

**Table 1:** Composition of RUTF formulations.

Blend A		Blend B		Blend C	
Ingredient	%	Ingredient	%	Ingredient	%
Soybean	30	Soybeans	30	Soybeans	30
Date palm	28	Brown sugar	28	Brown sugar	28
Peanuts	25	Soya oil	20	Soya oil	25
Soya oil	15	Rice	20	Wheat	15
Baobab	2	Baobab	2	Baobab	2
Total	100	Total	100	Total	100

**Table 2:** Proximate analysis of RUFT per 100 g.

Variable	Blend A (n = 3)	Blend B (n = 3)	Blend C (n = 3)	F(2,6)	*p*-Value	η^2^
Energy (kcal)	563.08 ± 00.25 ^a^	503.67 ± 00.12 ^b^	528.98 ± 00.27 ^c^	58,240.1	<0.001	0.99
Moisture (%)	1.34 ± 00.11 ^a^	2.49 ± 00.97 ^ab^	4.11 ± 00.89 ^b^	9.84	0.013	0.77
Protein (%)	13.56 ± 00.75 ^a^	16.71 ± 01.12 ^b^	14.62 ± 00.59 ^a^	11.83	0.008	0.80
Fat (%)	35.84 ± 00.71 ^a^	28.15 ± 00.18 ^b^	34.10 ± 02.64 ^a^	19.45	0.002	0.87
Ash (%)	1.27 ± 00.09 ^a^	3.76 ± 00.39 ^b^	2.64 ± 00.38 ^c^	48.33	<0.001	0.94
Carbohydrate (%)	46.57 ± 01.48 ^a^	45.87 ± 03.35 ^a^	40.90 ± 02.85 ^b^	3.98	0.080	0.57

Values are presented as mean ± standard deviations of triplicate readings, n = 3. Means with different superscripts (a, b, c) differ significantly at the Bonferroni-adjusted level (*p* < 0.017).

**Table 3:** Sensory evaluation results.

Attribute	Blend A	Blend B	Blend C	F(2,57)	*p*-Value	η^2^
Taste	6.70 ± 1.92	6.50 ± 1.87	7.00 ± 1.58	0.43	0.652	0.01
Flavour	5.33 ± 2.16	6.17 ± 2.48	6.17 ± 2.48	0.98	0.382	0.03
Aroma	7.00 ± 1.58	7.00 ± 1.59	6.00 ± 2.00	2.26	0.113	0.07
Appearance	6.00 ± 2.83	6.17 ± 2.48	5.73 ± 2.56	0.15	0.860	0.01
Overall Acceptability	5.50 ± 1.29 ^a^	7.50 ± 1.29 ^b^	5.33 ± 1.53 ^a^	14.12	<0.001	0.33

Values are presented as mean ± standard deviations of panellists’ scores, n = 20. Means with different superscripts (a, b) differ significantly at the Bonferroni-adjusted level (*p* < 0.017).

**Table 4:** Vitamin concentrations of the three RUTF blends.

Variable	Blend A	Blend B	Blend C	F(2,6)	*p*-Value	η^2^
Vitamin A (µM)	9.13 ± 00.02 ^c^	15.89 ± 00.37 ^a^	12.86 ± 00.15 ^b^	648.5	<0.001	0.99
Vitamin C (mg/100 g)	7.39 ± 00.10 ^c^	9.57 ± 00.24 ^b^	11.70 ± 00.14 ^a^	479.8	<0.001	0.99
Vitamin E (mg/100 g)	9.29 ± 00.19 ^a^	8.16 ± 00.19 ^b^	8.59 ± 00.12 ^c^	33.8	<0.001	0.92

Values are Mean ± Standard Deviation. Means with different superscripts (a, b, and c) within the same row are significantly different at the Bonferroni-adjusted level (*p* < 0.017).

**Table 5:** Mineral composition of the RUTF per 100 g.

Mineral (mg/100 g)	Blend A	Blend B	Blend C	F(2,6)	*p*-Value	η^2^
Zinc	0.35 ± 00.00 ^a^	0.39 ± 00.00 ^b^	0.31 ± 00.00 ^c^	1250.0	<0.001	0.99
Iron	0.45 ± 00.15 ^a^	2.40 ± 00.02 ^b^	1.05 ± 00.01 ^c^	302.4	<0.001	0.99
Potassium	2.70 ± 00.04 ^a^	2.94 ± 00.66 ^a^	61.65 ± 01.43 ^b^	4580.2	<0.001	0.99
Magnesium	1.07 ± 00.04 ^a^	0.99 ± 00.04 ^b^	0.85 ± 00.04 ^c^	28.5	0.001	0.90
Calcium	13.34 ± 00.11 ^a^	21.05 ± 00.04 ^b^	16.02 ± 00.08 ^c^	5822.1	<0.001	0.99
Sodium	6.04 ± 00.00 ^a^	6.99 ± 00.04 ^b^	6.21 ± 00.14 ^a^	88.6	<0.001	0.96

Values are presented as mean ± standard deviations of triplicate readings, n = 3. Means with different superscripts (a, b, and c) differ significantly at the Bonferroni-adjusted level (*p* < 0.017).

**Table 6:** Comparison with standard RUTF (WHO).

Nutrient Content	WHO RUTF	Blend A	Blend B	Blend C
Protein (g/100 g)	10–12%	13.56%	16.71%	14.62%
Energy (kcal/100 g)	520–550	563.08	503.67	528.98
Carbohydrate (g/100 g)	30–40%	46.57%	45.87%	40.90%
Lipids (g/100 g)	45–60%	35.84%	28.15%	34.10%
Ash (g/100 g)		1.27%	3.76%	2.64%
Moisture (g/100 g)	2.5%	1.34%	2.49%	4.11%
Magnesium (mg/100 g)	80–140	1.07	0.99	0.85
Calcium (mg/100 g)	300–785	13.34	21.05	16.02
Ca-Mg ratio	2:1	12.47	21.26	18.55
Iron (mg/100 g)	10–14	0.45	2.40	1.05
Zinc (mg/100 g)	11–14	0.35	0.39	0.31
Potassium (mg/100 g)	1100–1600	2.70	2.94	61.65

Source: RUTF figures adapted from UNICEF [21] and WHO [22].

## Data Availability

The original contributions presented in this study are included in the article. Further inquiries can be directed to the corresponding author.
